# The relationship between air pollutants and maternal socioeconomic factors on preterm birth in California urban counties

**DOI:** 10.1038/s41370-021-00323-7

**Published:** 2021-04-15

**Authors:** Zesemayat K. Mekonnen, John W. Oehlert, Brenda Eskenazi, Gary M. Shaw, John R. Balmes, Amy M. Padula

**Affiliations:** 1 University of California Berkeley-University of California San Francisco Joint Medical Program, Berkeley, CA USA; 2 Department of Pediatrics, Division of Neonatology, Stanford University, Stanford, CA USA; 3 School of Public Health, University of California Berkeley, Berkeley, CA USA; 4 Department of Medicine, University of California San Francisco, San Francisco, CA USA; 5 Department of Obstetrics, Gynecology and Reproductive Sciences, University of California San Francisco, San Francisco, CA USA

**Keywords:** Preterm birth, Air pollution, Socioeconomic status, Ozone, PM_2.5_

## Abstract

**Background:**

Preterm birth is the leading cause of perinatal morbidity and mortality in the U.S. and disparities among racial and ethnic groups persist. While etiologies of preterm birth have not been fully elucidated, it is probable that environmental and social factors play a role.

**Objective:**

We hypothesized that there is an interactive association between exposure to fine particulate matter (PM_2.5_) or ozone (O_3_) and neighborhood socioeconomic factors that increase the risk of preterm birth.

**Methods:**

We conducted a retrospective study using geocoded birth certificate data between 2007 and 2011, daily ambient air quality data on PM_2.5_ and O_3_, and American Community Survey (2007–2011 5-year estimates) data to assess census tract-level socioeconomic factors in California urban counties.

**Results:**

Our study found a small positive association between maternal exposures to PM_2.5_ and O_3_ and preterm birth that varied by gestational exposure period. In mixed-effects models, we found an increase in the risk of preterm birth for a one-unit change in PM_2.5_ averaged across the entire pregnancy (AOR = 1.02, 95% CI: 1.01, 1.02) and O_3_ during 3-months pre-pregnancy (AOR = 1.03, 95% CI: 1.02, 1.04). Interaction between census tract-level factors and air pollutants showed an increase in the risk of preterm birth among mothers living in higher socioeconomic areas, though, a fixed cohort bias sensitivity analysis showed these associations were not significant.

**Significance:**

These findings substantiate previous studies that showed associations between air pollution and preterm birth, even as pollution levels have decreased. This study has important implications for policy decisions and may help inform research on potential mechanisms of preterm birth.

## Introduction

Approximately 10% of all infants born in California are preterm [1]. Preterm birth is defined as birth at less than 37 weeks of gestational age and is the leading cause of perinatal morbidity and mortality in the United States [2]. It remains an important problem that has substantial public health costs to society in child follow-up care and future adverse health outcomes in adulthood [3, 4]. Children born prematurely are more likely to have developmental problems and learning disabilities such as cerebral palsy, impaired cognitive development, and sensory deficits as well as an increased risk of chronic disease in adulthood such as type 2 diabetes mellitus and cardiovascular disease [5].

While the pathophysiology of preterm birth is still being investigated, it is probable that a combination of genetic, social, and environmental factors contribute [6]. Since preterm birth is thought to be a condition induced by multiple pathways and heterogeneous mechanisms, it is difficult to identify intervention targets and achieve prevention results [7]. Risk factors for preterm birth include advanced maternal age, self-reported race and ethnicity, a plurality of births, co-morbidities, a prior preterm birth history, and environmental or social factors [8]. In particular, previous studies have shown that the use of self-reported race and ethnicity is a risk factor for preterm birth and approximates lived experiences of systemic discrimination and racism [9–13]. Evidence on the role of chronic exposure to stress includes the effect of “weathering” and the increased allostatic load of stress experienced by women of color as well as changes seen to the hypothalamic-pituitary axis and immune system that may lead to preterm birth [14–18]. While there is no established standard measure or instrument to quantify racism in epidemiological studies, self-reported race and ethnicity may be used as a proxy for many domains of racism and racial discrimination, particularly in studies without contact with subjects [19]. Several studies, after accounting for the individual socioeconomic characteristics of mothers, reported an association between measures of neighborhood socioeconomic deprivation and preterm birth [3, 20].

In conjunction with social factors, another important area of investigation is the role of environmental factors. Outdoor air pollution has been associated with an increased risk of morbidity and mortality associated with multiple diseases including cardiovascular disease, acute respiratory infection, asthma, and adverse pregnancy outcomes, including preterm birth [21]. Studies have shown that maternal exposure to air pollutants during pregnancy increases the risk of preterm birth through various processes related to inflammation, oxidative stress, endocrine disruption, and impaired oxygen transport across the placenta [22, 23]. In particular, several studies have found that fine particle matter with a diameter of less than or equal to 2.5 μm (PM_2.5_), is associated with preterm birth and adverse birth outcomes [21]. Padula et al. [24] investigated the relationship between exposure to traffic-related air pollution (TRAP) in the San Joaquin Valley during pregnancy and the risk of preterm birth [24]. They found increased odds of early preterm birth (i.e., delivery before 32 weeks gestation) in women exposed to the highest quartile of TRAP during the second trimester providing evidence in support of an air pollution-preterm birth association that has been reported by other investigators [25]. In addition, other studies conducted in the U.S. and globally have shown associations between ozone (O_3_) and preterm birth [26, 27].

Moreover, exposure to air pollution is disproportionately higher in communities of color and lower socioeconomic status (SES) [28, 29]. Combined with the health inequities seen in preterm delivery among Black births, a reproductive and environmental justice framework is useful when studying the relationship between exposure to air pollutants and sociodemographic factors on preterm birth and its implications for policy [30, 31]. Environmental justice refers to the fair and equal treatment and right to a clean environment [32]. Pursuant to the Clean Air Act, the U.S. environmental protection agency (EPA) established a network of air pollution monitors in order to assess whether areas were in compliance with the nationally set ambient air quality standards. The use of air quality measurements from these monitors in combination with sociodemographic information on preterm birth can be an important way to assess if the current standards are unmet or inadequate [33, 34]. Several studies have shown stronger associations of preterm birth with exposure to air pollutants when interaction with neighborhood-level sociodemographic covariates is included in the analysis [35–37]. Reproductive justice refers to the right to have children or not and parent those children in a safe and healthy environment [38]. This intersectionality with environmental justice is an important rationale for investigating the relationship between exposure to air pollutants such as PM_2.5_ and O_3_ and social factors during pregnancy and preterm birth. We sought to determine if there is an interactive association between exposure to air pollutants and neighborhood socioeconomic factors that increased the risk of preterm birth in California urban counties during 2007–2011. Understanding etiologies of preterm birth including both racial/ethnic and environmental factors and their possible interaction may help inform policy decisions to address some of the health disparities that currently exist.

## Methods

### Study population

Birth certificates from all births to women living in the six California counties of Alameda, Contra Costa, Fresno, Los Angeles, San Diego, and San Francisco from 2007 to 2011 were obtained from the Birth Statistical Master files at the California Department of Public Health. The study population originated from 1,132,953 births from these six counties between 2007 and 2011 (Supplemental data, Fig. S1). Exclusions for the study population included those with multiple births, i.e., twins or greater (*n* = 36,160), those with gestational age missing or less than 20 weeks or more than 42 weeks (*n* = 11,578), missing covariates (maternal age, education, race/ethnicity) (*n* = 49,169). All remaining birth records were merged with air pollution exposure data for O_3_ and PM_2.5_ (*n* = 68,394 did not have available air pollutant data). Finally, births without complete exposure defined as availability estimates for 3 months pre-pregnancy and at least 5 months post-conception were excluded (*n* = 13,701). The final study population included 953,951 singleton births.

The maternal residence at birth locations, street addresses, obtained from California birth certificates were geocoded to *x* and *y* coordinates with ArcGIS software (ESRI, Redlands, CA) and residence addresses were corrected with ZP4 software (Semaphore Corporation, Aptos, CA). Maternal factors from the Birth Statistical Master files included: maternal age, maternal race and ethnicity, maternal education, maternal country of origin, source of payment for birth delivery (Medi-Cal, private, uninsured, other), number of prenatal care visits, month prenatal visits began, year of birth, the season of conception, and self-reported tobacco use. Additional data on current smoking status, prenatal care, maternal pre-pregnancy, and gestational diabetes and hypertension, maternal height, and pre-pregnancy weight were obtained from the hospital discharge record obtained through the office of statewide health planning and development. These data were linked to birth statistical master files using a probability matching algorithm that included infant birth date, birth hospital, delivery mode, and mother’s date of birth, and other maternal information [39, 40]. All protocols for the study were approved by the Committee for the Protection of Human Subjects within the Health and Human Services Agency of the State of California and the Institutional Review Boards of the University of California, San Francisco, and Stanford University.

We used the 2007–2011 5-year American community survey to examine census tract-level variables of SES: the GINI index of income inequality, highest educational attainment for populations 25 years and older, percent unemployed population in the labor force for populations 25 years and older, median household income in dollars, the percent at poverty level, and households with public assistance income. These variables were used to capture area-level socioeconomic factors of an individual’s surroundings. The boundaries do not necessarily correspond to where individuals spend their time, but the census tract for this purpose is a proxy for the neighborhood.

Ambient air quality data for daily ozone (O_3_) and fine particulate matter (PM_2.5_) were obtained from the national air monitoring stations/state and local air monitoring stations. The EPA developed a Bayesian space-time fusion model to estimate daily 8-h maximum O_3_ concentration and 24-h average of PM_2.5_ for each 2010 US census tract centroid in the United States. The model fuses data from the ground-based monitoring network with a community model for air quality model estimates with output on 12*12 km grids [41]. Data for ambient air quality data for daily O_3_ and PM_2.5_ were summarized into 1-month exposure periods backward from live birth until a 12-month exposure period had been estimated. They were then further pooled into 3-month or trimester estimates. Air pollutant measurements were dichotomized into high and low categories. The cutoff PM_2.5_ was set by the median, 12.9 µg/m^3^, which is slightly higher than the annual EPA limit of 12 µg/m^3^ [42]. For O_3_, the cutoff was set by the median, 39 ppb, which is substantially lower than the EPA 8-h max of 0.070 ppm [43].

Data from air pollution estimates and socioeconomic factors were merged at the census tract level and birth records were assessed at the individual level.

### Statistical methods

#### Primary analysis

Statistical analyses were performed using the R statistical program in RStudio (RStudio, Inc, Boston, MA). We used logistic regression models to estimate odds ratios (OR) and 95% confidence intervals (CI) to quantify the risk of total preterm birth (<37 weeks gestation) and early preterm birth (<32 weeks gestation) at the individual level in relation to a 1-unit change in pollutant (PM_2.5_ and O_3_). Two-group comparisons for continuous parametric variables were made utilizing the Student *t*-test and a Wilcoxon rank-sum for non-parametric continuous variables. Two-group comparisons for dichotomous variables were made using a Pearson’s chi-square test. The correlation was calculated using Pearson’s correlation coefficients. According to our a priori analysis plan, each model was adjusted for the following covariates: maternal age (<20 and >35 years), race/ethnicity, education, prenatal care in the first trimester, cigarette use in the first trimester, year of birth, and insurance payment of delivery. Stratified analyses were conducted and tested for statistical multiplicative interaction using interaction terms between air pollution and census tract-level covariates (poverty, GINI index, education, public assistance, unemployed, median HH income). Census tract-level covariates were placed into dichotomous high and low categories. Then, each model included a single individual SES factor with a single pollutant as an interaction term. Wald chi-squared tests were calculated for the interaction terms (pollutant*individual SES factor) to determine which census tract-level SES variables had multiplicative interactions with air pollution to affect preterm birth.

#### Secondary analysis

Additional sensitivity analyses were conducted to validate our primary findings. Mixed-effects models were generated using census tract to account for spatial autocorrection of study subjects. To address potential fixed cohort bias, we performed a sensitivity analysis wherein we removed births with conception dates 20 weeks or more before the beginning of the cohort and 42 weeks or less before the cohort ended [44]. This excluded 8% of births.

## Results

Demographic characteristics of the study population are shown in Table 1. The prevalence of preterm birth was 9.2% and differed among racial groups with the highest prevalence among Black women (12.9%). Preterm births were higher among mothers aged <20 or >35 years old. About a quarter of the population had a college degree or higher (26.5%) and roughly half of the individuals were insured by Medi-Cal (48.6%). Over the study period, there was a slight decrease in the frequency of preterm birth per year.

**Table 1:** Characteristics of the study population for singleton births of six counties in California, 2007–2011 *N* = 953,951.

	Preterm birth		Term birth	
	*N* = 87, 495 (9.2%)		*N* = 866,456 (90.8%)	
Maternal age (years), Mean ± SD *n* (%)	28.6 ± 6.7		28.5 ± 6.3	
15–20	8756 (10.6)		73,654 (89.4)	
20–25	17,881 (9.2)		175,949 (90.8)	
25–30	20,925 (8.5)		225,828 (91.5)	
30–35	21,049 (8.5)		226,455 (91.5)	
>35	18,884 (10.3)		164,570 (89.7)	
Race, *n* (%)				
White	43,451 (8.6)		462,365 (91.4)	
Black	8860 (12.9)		59,969 (87.1)	
Native American/Alaskan Native	297 (9.3)		2913 (90.7)	
Asian/Pacific Islander	9530 (8)		109,758 (92)	
Other/unknown	25,357 (9.9)		231,451 (90.1)	
Ethnicity, *n* (%)				
Hispanic (1)	48,176 (9.7)		448,935 (90.3)	
Non-Hispanic^a^ (2)	39,319 (8.6)		417,521 (91.4)	
Maternal origin of birth, *n* (%)				
Mother, foreign born	39,765 (8.9)		407,056 (91.1)	
Mother, US born	47,709 (9.4)		459,136 (90.6)	
Maternal education, *n* (%)				
Less than high school	25,976 (10.6)		217,999 (89.4)	
High school/GED	23,703 (9.9)		215,704 (90.1)	
Some college	20,320 (9.3)		197,219 (90.7)	
College degree or more	17,496 (6.9)		235,534 (93.1)	
Maternal health, *n* (%)				
Pre-existing hypertension	1699 (35.4)		3107 (64.6)	
No history of pre-existing hypertension	85,796 (0.9)		863,349 (90.1)	
Gestational hypertension	1640 (17.3)		7857 (82.7)	
No gestational hypertension	85,855 (0.9)		858,599 (90.1)	
Pre-existing diabetes	1721 (22.1)		6061 (77.9)	
No history of pre-existing diabetes	85,774 (0.9)		860,395 (90.1)	
Gestational diabetes	1824 (21.6)		6627 (78.4)	
No gestational diabetes	85,671 (0.9)		859,829 (90.1)	
Pre-eclampsia	7885 (28.3)		19,931 (71.7)	
No pre-eclampsia	79,610 (0.9)		846,525 (90.1)	
Tobacco use, *n* (%)				
Non-smokers	83,417 (0.9)		839,703 (90.1)	
>0 and <10 cigs/day	1386 (13.8)		8662 (86.2)	
10–20 cigs/day	51 (12.0)		374 (88.0)	
>20 cigs/day	250 (14.8)		1433 (85.2)	
BMI (pre-pregnancy), M ± SD	26.2 ± 6.3		25.6 ± 6.1	
Number of Prenatal care visits, M ± SD	11.2 ± 4.5		12.4 ± 3.6	
Insurance at time of delivery, *n* (%)				
Medi-Cal	47,685 (10.3)		415,961 (89.7)	
Private	36,247 (8)		417,674 (92)	
Birth year, *n* (%)				
2007	19,561 (9.9)		178,714 (90.1)	
2008	17,400 (9.5)		165,718 (90.5)	
2009	16,690 (9.3)		162,154 (90.7)	
2010	17,302 (8.7)		181,466 (91.3)	
2011	16,542 (8.5)		178,404 (91.5)	
Season of conception, *n* (%)				
Fall	22,211	(9.3)	217,853	(90.7)
Winter	21,740	(9)	218,864	(91)
Spring	21,736	(9.1)	215,990	(90.9)
Summer	21,808	(9.3)	213,749	(90.7)
Parity, *n* (%)				
Nulliparous	33,587	(8.6)	357,594	(91.4)
1 or more	53,908	(9.6)	508,862	(90.4)
Sex of infant, *n* (%)				
Female	39,340	(8.5)	425,275	(91.5)
Male	48,150	(0.8)	441,180	(90.2)
Unknown	5	(83.3)	1	(16.7)
Exposure to PM_2.5_ (µg/m^3^), whole pregnancy, M ± SD	13.5 ± 3.1		13.2 ± 2.9	
Exposure to O_3_ (ppb), whole pregnancy, M ± SD	39.0 ± 7.6		38.8 ± 6.8	

^a^Included unknown/other.

Characteristics of the study population were compared across maternal exposure to air pollutants, PM_2.5_ and O_3_ (Table S1). Mothers with an “other” or unknown race were more likely to live in areas with higher PM_2.5_ and White mothers were more likely to live in areas with higher O_3_. Owing to the decrease of PM_2.5_ over the study period, the number of births in the high PM_2.5_ category diminished from 2007 (31.5%) to 2011 (2.3%).

Census tract-level covariates were compared between preterm and term births (Table 2). Among preterm births, the average median household income of the census tract of maternal residence was lower ($55,716) than among term births ($59,947). All socioeconomic factors were statistically different between preterm and term birth census tracts except for the GINI index of income inequality. We calculated a correlation matrix for the census tract-level covariates, which revealed strong correlations between the poverty, income, education, and state and federal assistance programs (SNAP/CalFresh, a food assistance program, public assistance—only including general assistance and temporary assistance to needy families (TANF) assistance, and total assistance—including all programs) (Fig. S2).

**Table 2:** Bivariate analysis between neighborhood census-tract covariates and birth outcome.

	Preterm births	Term births	*p*-value
	*N* = 87,495 (9.2%)	*N* = 866,456 (90.8%)	
*Neighborhood census tract-level variables, Mean* *±* *SD*			
Median household income, dollars	55,716 ± 26,577	59,497 ± 28,216	<0.001
Percent of population with income below poverty level	19.2 ± 12.9	17.5 ± 12.3	<0.001
GINI index of income inequality	0.408 ± 0.06	0.408 ± 0.06	0.18
Percent of population that graduated high school^*^	72.9 ± 18.9	75.4 ± 18.8	<0.001
Percent total population unemployed^*^	35.3 ± 7.2	34.8 ± 7.2	<0.001
Percent on total assistance (include SSI, SNAP, Public assistance)	23.4 ± 17.3	21.1 ± 16.7	<0.001
Percent on public assistance	5.4 ± 5.1	4.8 ± 4.7	<0.001
Percent on SNAP/food stamps	11.1 ± 9.4	9.8 ± 8.9	<0.001
Percent on supplemental security income	6.5 ± 4.4	6.1 ± 4.3	<0.001

*SSI* supplemental security income, *SNAP* supplemental nutritional assistance program.

^*^Population 25 years and older; *P*-value is from Chi-Squared Pearson test.

### Preterm birth and air pollution

**Fig. 1:**
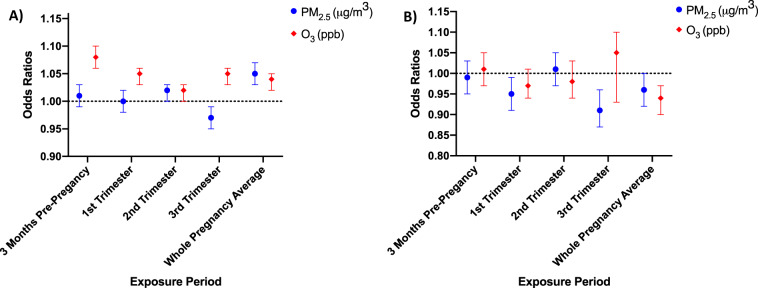
Associations between air pollutants, PM_2.5_ and O_3_, and preterm birth by maternal exposure period. Adjusted odds ratio (aOR) included a season of conception, cigarette use, age, race/ethnicity, payment, prenatal visits that began in the first trimester. **A** For preterm birth (<37 weeks), the aOR for all exposure periods was greater than one. For O_3_ exposure, the aOR was highest in the 3-month pre-pregnancy period for every 1-unit (ppb) increase in O_3_ (aOR = 1.10, 95% CI: 1.08, 1.12), while for PM_2.5_, the highest aOR of preterm birth was associated during the whole pregnancy exposure period (aOR = 1.10, 95% CI: 1.09, 1.12). **B** For early preterm birth (<32 weeks), an increased risk was associated with O_3_ during the whole pregnancy period (aOR = 1.07, 95% CI: 1.03, 1.11) and PM_2.5_ during the third trimester (aOR = 1.09, 95% CI: 1.04, 1.14).

Table 3 presents the crude and adjusted ORs estimating the associations between maternal exposure to air pollutants with total preterm birth (<37 weeks) and early preterm birth (<32 weeks). We found a positive association between both PM_2.5_ and O_3_ and preterm birth (Fig. 1). We saw the highest risk of preterm birth associated with a 1-unit (μg/m^3^) increase in exposure to PM_2.5_ during the whole pregnancy exposure period [adjusted odds ratio (AOR) = 1.05, 95% CI: 1.03, 1.07], whereas we found the association between O_3_ and preterm birth to be highest in the 3-month pre-pregnancy period for every 1-unit (ppb) increase in O_3_ (AOR = 1.08, 95% CI: 1.06, 1.10). For early preterm birth (<32 weeks), we found the highest risk associated with O_3_ during the third trimester pregnancy period (AOR = 1.05, 95% CI: 0.93, 1.10) and no significant association for PM_2.5_ during any period.

**Table 3:** Association between maternal exposure to PM_2.5_ and O_3_ with preterm birth and early preterm birth by exposure period.

	Preterm birth (<37 weeks)		Early preterm birth (<32 weeks)	
	*N* = 87,495/953,951		*N* = 15,418/87,495	
	Crude	Adjusted^a^	Crude	Adjusted^a^
Exposure to PM_2.5_	COR (95% CI)	AOR^a^ (95% CI)	COR (95% CI)	AOR^a^ (95% CI)
3 months pre-pregnancy	1.10 (1.08, 1.12)	1.01 (0.99, 1.03)	1.01 (0.97, 1.05)	0.99 (0.95, 1.03)
First trimester	1.10 (1.09, 1.12)	1.00 (0.98, 1.02)	1.04 (1.00, 1.08)	0.95 (0.91, 0.99)
Second trimester	1.12 (1.10, 1.13)	1.02 (1.00, 1.03)	1.00 (0.97, 1.04)	1.01 (0.97, 1.05)
Third trimester	1.08 (1.07, 1.10)	0.97 (0.95, 0.99)	1.07 (1.03, 1.11)	0.91 (0.87, 0.96)
Whole pregnancy	1.16 (1.14, 1.17)	1.05 (1.03, 1.07)	1.04 (1.01, 1.08)	0.96 (0.92, 1.00)
*Exposure to O*_*3*_				
3 months pre-pregnancy	1.07 (1.05, 1.08)	1.08 (1.06, 1.10)	1.00 (0.96, 1.03)	1.01 (0.97, 1.05)
First trimester	1.05 (1.03, 1.06)	1.05 (1.03, 1.06)	1.03 (1.00, 1.07)	0.97 (0.94, 1.01)
Second trimester	1.01 (1.00, 1.03)	1.02 (1.00, 1.03)	1.01 (0.98, 1.05)	0.98 (0.94, 1.03)
Third trimester	1.04 (1.02, 1.05)	1.05 (1.03, 1.06)	1.02 (0.98, 1.07)	1.05 (0.93, 1.10)
Whole pregnancy	1.00 (0.98, 1.01)	1.04 (1.02, 1.05)	1.08 (1.05, 1.12)	0.94 (0.90, 0.97)

^a^Adjusted for a season of conception, maternal cigarette use, age, race/ethnicity, education, payment, prenatal visits began in the first trimester, year of birth.

^b^High/low cutoff is median PM_2.5_ = 12.9, high/low cutoff for is median O_3_ = 39 ppb for the whole pregnancy (EPA limits are Annual PM_2.5_ = 12 µg/m^3^, 8-h max O_3_ = 0.070 ppm) include the reference category. High is the reference category.

Associations between pollutants and preterm birth differed by the season of conception (Table S4). Births conceived in the spring (March–May) compared to other seasons had larger associations between O_3_ and preterm birth with AORs ranging between 1.03 and 4.48 across the different exposure periods. Births conceived in the summer (June–August) compared to other seasons had stronger associations between PM_2.5_ and preterm birth with AORs ranging between 1.06 and 1.41 across the different exposure periods. Overall, the remaining seasons of conception had associations that were less consistent and sometimes in opposite directions with each pollutant.

We examined whether the relationship between air pollutants and preterm delivery differed by various measures of census tract-level SES during the entire pregnancy period for total preterm and early preterm birth (Table 4). We found that mothers who lived in areas with a high GINI index, a marker of income inequality, and were exposed to higher levels of PM_2.5_ during the whole pregnancy had increased risk of total preterm birth per 1-unit μg/m^3^ (AOR = 1.04, 95% CI: 1.01, 1.07: Table 4) than mothers who lived in lower GINI index areas (AOR = 0.97, 95% CI: 0.94, 0.99; Table 4). However, when we stratified the analysis for early preterm birth, the association between whole pregnancy exposure to PM_2.5_ and GINI index was no longer significant. In subsequent analyses of early preterm birth by exposure period, we observed that the only significant factor associated with an increased risk occurred for areas with high unemployment and high exposure to PM_2.5_ in the 3-month pre-pregnancy period (AOR = 1.08, 95% CI: 1.01, 1.17; Table S3) compared to low unemployment (AOR = 0.92, 95% CI: 0.86, 0.99; Table S3). Several censuses tract-level markers of low SES in conjunction with high O_3_ exposure during pregnancy were associated with increased risk of total preterm birth: low household income, low education, high total assistance, high supplemental nutritional assistance program (SNAP), high public assistance, high supplemental security income (SSI) and high poverty (AORs ranged from 1.04 to 1.11; Table 4) when compared to census tracts with higher SES (AORs ranged from 0.90 to 0.97; Table 4). However, for early preterm birth and O_3_ exposure, we found a reversal with census tracts characterized by higher SES associated with increased risk of preterm birth: high household income, high education, low public assistance, low employment, and low poverty (AORs ranged from 1.07 to 1.10; Table 4) compared to census tracts with lower SES (AORs ranged from 0.91 to 0.93; Table 4). In analyses by exposure period, O_3_ during the 3 months pre-pregnancy was associated with an increased risk of total preterm birth with high total assistance and high poverty (AOR = 1.03, 95% CI: 1.00, 1.06; Table S2) and a larger risk of total preterm birth with high GINI (AOR = 1.06, 95% CI: 1.03, 1.09; Table S2) compared to areas of low total assistance, low poverty, and low GINI. High O_3_ exposure during the third trimester and low SES factors including low education, high total assistance, and high SNAP were associated with early preterm birth (AORs ranged from 1.09 to 1.13; Table S3) compared to areas with high education, low total assistance, and low SNAP (AORs ranged from 0.88 to 0.90; Table S3).

**Table 4:** Interaction between whole pregnancy average maternal exposure to PM_2.5_ and O_3_ and neighborhood census tract-level covariates on preterm birth and early preterm birth (aOR^a^ ± 95%CI).

	Preterm birth (<37 weeks)			Early preterm birth (<32 weeks)		
	High SES	Low SES		High SES	Low SES	
Exposure to PM_2.5_	aOR^a^ (95% CI)	aOR^a^ (95% CI)	*p*-value	aOR^a^ (95% CI)	aOR^a^ (95% CI)	*p*-value
Household Income ($)	1 (0.91, 1.03)	1 (0.97, 1.03)	0.63	1 (0.92, 1.06)	1 (0.94, 1.09)	0.75
High school grad (%)	1 (1.00, 1.06)	1 (0.94, 1.00)	**0.03**	1 (0.91, 1.05)	1 (0.95, 1.10)	0.58
Total assistance (%)	1 (0.97, 1.03)	1 (0.97, 1.03)	0.93	1 (0.95, 1.10)	1 (0.91, 1.05)	0.56
SNAP (%)	1 (1.00, 1.06)	1 (0.95, 1.00)	0.06	1 (0.91, 1.05)	1 (0.95, 1.10)	0.56
Public assistance (%)	1 (1.00, 1.06)	1 (0.94, 1.00)	**0.05**	1 (0.91, 1.05)	1 (0.96, 1.10)	0.47
SSI (%)	1 (0.98, 1.04)	1 (0.96, 1.02)	0.41	1 (0.94, 1.06)	1 (0.92, 1.06)	0.75
Poverty (%)	1 (0.98, 1.04)	1 (0.96, 1.02)	0.48	1 (0.90, 1.04)	1 (0.96, 1.11)	0.44
Unemployment (%)	1 (1.00, 1.06)	1 (0.94, 1.00)	**0.04**	1 (0.94, 1.08)	1 (0.92, 1.07)	0.85
GINI Index (%)	1 (0.94, 0.99)	1 (1.01, 1.07)	**0.02**	1 (0.93, 1.07)	1 (0.93, 1.08)	0.93
*Exposure to O*_*3*_						
Household income ($)	0.9 (0.91, 0.97)	1.1 (1.03, 1.10)	**<0.001**	1.1 (1.02, 1.18)	0.9 (0.84, 0.98)	**0.009**
High school grad (%)	0.9 (0.92, 0.97)	1.1 (1.03, 1.09)	**<0.001**	1.1 (1.01, 1.16)	0.9 (0.86, 0.99)	**0.03**
Total assistance (%)	0.9 (0.90, 0.95)	1.1 (1.05, 1.11)	**<0.001**	1.1 (1.02, 1.17)	0.9 (0.85, 0.98)	**0.02**
SNAP (%)	0.9 (0.88, 0.93)	1.1 (1.08, 1.14)	**<0.001**	1.1 (0.99, 1.15)	0.9 (0.87, 1.01)	0.08
Public assistance^b^ (%)	0.9 (0.88, 0.93)	1.1 (1.08, 1.14)	**<0.001**	1.1 (1.01, 1.16)	0.9 (0.86, 0.99)	**0.03**
SSI (%)	1 (0.94, 0.99)	1 (1.01, 1.07)	**0.02**	1 (0.97, 1.12)	1 (0.89, 1.03)	0.25
Poverty (%)	1 (0.94, 0.99)	1 (1.01, 1.07)	**0.01**	1.1 (1.02, 1.18)	0.9 (0.85, 0.98)	**0.01**
Unemployment (%)	1 (0.98, 1.03)	1 (0.97, 1.02)	0.69	1.1 (1.00, 1.15)	0.9 (1.00, 1.06)	**0.05**
GINI index (%)	1 (0.97, 1.03)	1 (0.97, 1.03)	0.89	1 (0.92, 1.06)	1 (0.94, 1.09)	0.69

Bold values indicate statistical significance *p* < 0.05.

Air pollution is a dichotomous variable, the cutoff is median PM_2.5_ = 12.9, high/low cutoff is median O_3_ = 39 ppb for the whole pregnancy (EPA limits are Annual PM_2.5_ = 12 µg/m^3^, 8-h max O_3_ = 0.070 ppm) include the reference category. Low is the reference category.

High/low SES is defined by above vs. below the median for each socioeconomic factor.

*HH* household, *HS* high school, *Assistance* Total assistance, *SNAP* supplemental nutrition assistance program, *SSI* supplemental security income, *GINI* GINI index for income inequality.

^a^Adjusted for a season of conception, maternal cigarette use, age, race/ethnicity, education, payment, prenatal visits began in the first trimester, median used for high/low cut-off of census tract-level covariates; *P*-value is from Wald Chi-Squared test.

^b^ Public assistance includes general assistance and temporary assistance to needy families (TANF).

We completed additional analysis to adjust for spatial autocorrection of study participants with mixed-effects models. We found a smaller association between our previous findings where whole pregnancy exposure to PM_2.5_ was associated with increased odds of preterm birth (AOR = 1.02, 95% CI: 1.01, 1.02; Table S5), while high O_3_ exposure in the 3 months pre-pregnancy period was associated with increased odds of preterm birth (AOR = 1.03, 95% CI: 1.01, 1.02). In addition, we also performed a fixed cohort sensitivity analysis that showed a reversal of the PM_2.5_ findings. This analysis showed no significant association between PM_2.5_ and preterm birth. For O_3_, we found the highest association for preterm birth was in the third trimester pregnancy period (AOR = 1.11, 95% CI:1.10, 1.15; Table S6).

## Discussion

Our study showed a positive association between maternal exposure to both PM_2.5_ and O_3_ and preterm birth that is consistent with previous research studies. Our California study is one of the few studies to investigate the relationship between air pollutants and preterm birth with both individual-level and census tract-level markers of SES among urban counties. Our study is also notable that our data were collected for recent years during which air pollution in California has continued to decrease [45].

We found that maternal exposures to PM_2.5_ and O_3_ were associated with increased odds of preterm birth and that associations varied by gestational timing of exposure. While prior studies have observed trimester-specific associations between preterm birth and air pollutants, the results have been mixed. Our analysis also incorporated an additional exposure window, 3 months prior to pregnancy. While we had initially observed that higher exposure to PM_2.5_ had its largest associations with the second trimester and whole pregnancy exposure periods, these associations were inconsistent in additional sensitivity analyses we completed using mixed-effects modeling and fixed cohort bias analysis. The association between exposure to PM_2.5_ and preterm birth during the whole pregnancy exposure period remained significant for the mixed-effects modeling but was no longer significant with the fixed cohort analysis. A study by Sheridan et al. [46] discusses this complexity in results and suggests that the use of shorter exposure windows may have a higher sensitivity to establishing a critical period for PM_2.5_ and preterm birth [46]. Another possible explanation for the null results in the fixed cohort analysis is the beneficial impact from regulations passed by the California Air Resources Board. In particular, the California Truck and Bus Regulation promulgated in 2008 reduced toxic air contaminants from heavy-duty diesel vehicles, of which PM_2.5_ is included [47, 48]. We saw an overall reduction in preterm births over our study period that may correlate with the impact of the California Truck and Bus Regulation. Future studies are needed to fully explore the impact this regulation has had on preterm birth.

Interestingly, we found mothers exposed to higher levels of O_3_ during the 3 months prior to pregnancy exhibited the largest risk of preterm birth. This may be due to the inverse relationship between O_3_ and traffic-related air pollutants such as nitrogen oxides and PM_2.5_; however, it also suggests that critical periods surrounding conception and prior to pregnancy should be explored further. In addition, we saw different associations during critical periods of exposure to air pollutants for total preterm birth (<37 weeks) and early preterm birth (<32 weeks). Increased risk of early preterm birth was associated with high PM_2.5_ during the third trimester and high O_3_ over the whole pregnancy. This heightened risk of PM_2.5_ with early preterm birth during the third trimester was corroborated by our fixed cohort sensitivity analysis and has been shown in other studies. It may be due to the shortened third trimester period indicating possible overlap with the third trimester exposure period or may be due to the lower power in the subset data [46]. However, O_3_ exposure averaged over the entire pregnancy does not provide as much explanatory information is given seasonal variation of O_3_ throughout the year suggesting that another process may be underlying this finding.

Furthermore, we observed different associations with exposures to air pollutants when analyzed by the season of conception, which has also been seen in other studies. Results from these prior studies have been mixed with some showing increased odds of preterm birth for those conceived in autumn and others showing an increased odds of preterm birth for those conceived in summer [49]. Our results showed an increased risk of preterm birth for individuals conceived in the summer across all gestational exposure periods for PM_2.5_. This finding is supported by the increased odds of preterm birth for mothers with high levels of exposure to PM_2.5_ during the second and third trimester given that peak PM_2.5_ is seen during the winter months and this would correlate with a summer season of conception. For O_3_, we saw the greatest association with those individuals who were conceived in the spring, also correlating with increased sunlight exposure [50]. However, caution should be used in interpreting the higher OR in the third trimester given the varying lengths of the cases and controls are seen secondary to the inherent nature of preterm birth, i.e., those gestations that end in preterm have by definition shorter opportunity to be exposed than term gestations. While additional seasonal factors such as variation in temperature or infection rates could play a role, we do not believe these other factors are the primary drivers of these findings. Further studies are needed to disentangle the strong differences found by season.

Previous studies have suggested that neighborhood deprivation can modify the relationship between air pollution and preterm birth [37, 51, 52]. In our study, we found evidence of effect modification and showed larger associations between PM_2.5_ (third trimester and entire pregnancy exposure periods) and preterm birth in areas with high inequality (as measured by the GINI index) compared to areas of low inequality. Interestingly, we saw even more consistent effect modification of several census tract socioeconomic factors for the O_3_ exposure group. For the 3-month pre-pregnancy period, we saw positive associations for total preterm birth in mothers who lived in census tracts with a high percent of total assistance, a high percent of poverty, and a high GINI index. Given the positive association between O_3_ during the 3 months pre-pregnancy and preterm birth, this analysis may suggest that individuals with these markers of social deprivation are more vulnerable to the adverse effects of air pollution. In addition, we saw larger associations for almost all census tract-level factors and maternal exposure to O_3_ during the whole pregnancy for total preterm birth. Interestingly, when examining early preterm birth, we observed a higher risk of early preterm birth associated with higher exposure to O_3_ and higher levels of SES; however, the results should be taken in the context of the multiple comparisons of our secondary analyses. It is unclear what is driving this increase and it is possible that this finding was due to chance. We believe the further analysis is warranted.

These and previous epidemiological findings indicate that a deeper mechanistic understanding of the associations between exposures to air pollutants and preterm birth would be useful. Studies that have explored mechanistic underpinnings suggest that exposures to air pollutants, including PM_2.5_ and O_3_, result in higher levels of reactive oxygen species and increased markers of oxidative stress that may lead to adverse birth outcomes [53]. Another study looked at the role of biomarkers to elucidate the relationship between air pollution and preterm birth [54]. The authors showed that biomarkers for inflammation such as C-reactive protein and oxidative stress increased, while telomere length decreased and markers for epigenetic modification such as DNA methylation were varied for individuals exposed to increasing levels of air pollutants [54]. Although this research is still preliminary, it offers a promising area of future research that may help alert clinicians to better monitor women who are at increased risk of preterm birth during their pregnancy.

Strengths of our study include the large sample size and distribution of regions across the state of California, use of an innovative air quality model, and detailed individual-level covariates such as maternal cigarette use, pre-pregnancy BMI, and maternal comorbidities such as diabetes and hypertension that allowed us to evaluate the impact of these factors.

Our study had several limitations. Misclassification bias could have occurred when assigning air pollution exposure estimates. We used maternal residence at delivery as a proxy for the residential location for the entire pregnancy and pre-pregnancy period. However, it is possible that individuals moved during these times, previous research has shown that residential mobility is unlikely to have substantially affected the associations we observed [55, 56]. In addition, we assumed that the pollutant levels were spatially homogeneous within the 12-km grid and did not account for a finer resolution of pollutant gradients that may have existed as well as exposures that could have taken place at the workplace, during commutes, and while participating in an outside activity. However, this measurement error would likely have led to an underestimation of our results biasing them towards the null. Furthermore, the California Truck and Bus Regulation adopted in December 2008 aimed to reduce man-made toxic air contaminants (including PM_2.5_) emissions from heavy-duty diesel vehicles that may have influenced the reduction in air pollution and could partially explain the decrease in preterm birth seen over our study period [47, 48]. Lastly, while we were able to include numerous individual-level covariates in our study, some of those are self-reported. For example, maternal cigarette use was self-reported and could possibly be underreported in our dataset.

## Conclusion

Our study adds to the growing body of literature on the interactive impacts of socioeconomic factors and exposures to air pollution on preterm birth. Our findings are consistent with previous work on the associations between exposures to air pollution and preterm birth in California, with detailed air pollution exposure data and a large study population. It is notable that these associations with preterm birth persist even as the levels of these pollutants have recently decreased in California compared to earlier studies. These findings provide further evidence for the need for reproductive justice policies that can mitigate the impact exposures to air pollution have on the physical environment of under-resourced and low-income communities who have a higher burden of preterm birth.

## Supplementary information

SupFigure 1

SupFigure 2

SupTable 1

SupTable 2

SupTable 3

SupTable 4

SupTable 5

SupTable 6
