# Supplementary material for: The relationship between air pollutants and maternal socioeconomic factors on preterm birth in California urban counties
Source: J Expo Sci Environ Epidemiol. 2021 Apr 15;31(3):503–13. doi: 10.1038/s41370-021-00323-7 (PMC8134052; doi:10.1038/s41370-021-00323-7)
Supplement: Supplementary file 1 — SupFigure 1 [file 41370_2021_323_MOESM1_ESM.docx]

Figure S1. Flowchart of Final Study Population

*complete PM_2.5_ and O_3_ exposure data defined as having 3 month pre-pregnancy and at least 5 months post conception
