# Supplementary figures and images for: The relationship between air pollutants and maternal socioeconomic factors on preterm birth in California urban counties
Source: J Expo Sci Environ Epidemiol. 2021 Apr 15;31(3):503–13. doi: 10.1038/s41370-021-00323-7 (PMC8134052; doi:10.1038/s41370-021-00323-7)

Figure S2. Pearson correlation matrix of neighborhood census-tract level covariates


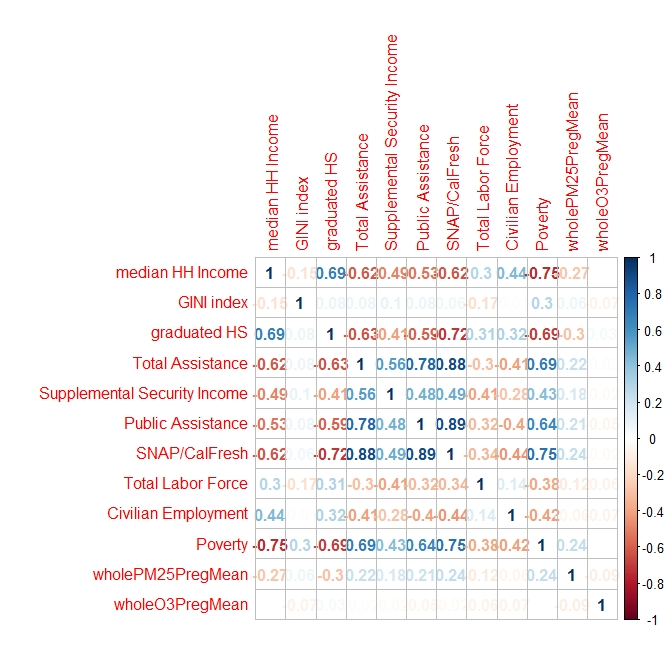

Supplement: Supplementary file 2 — SupFigure 2 [file 41370_2021_323_MOESM2_ESM.docx]
