# Supplementary material for: The relationship between air pollutants and maternal socioeconomic factors on preterm birth in California urban counties
Source: J Expo Sci Environ Epidemiol. 2021 Apr 15;31(3):503–13. doi: 10.1038/s41370-021-00323-7 (PMC8134052; doi:10.1038/s41370-021-00323-7)
Supplement: Supplementary file 3 — SupTable 1 [file 41370_2021_323_MOESM3_ESM.docx]

| Table S1. Characteristics of study population for singleton births by pollutant exposures in California, 2007-2011 N=953,951 | | | | | | | |  |
| --- | --- | --- | --- | --- | --- | --- | --- | --- |
|  |  |  |  |  |  |  |  |  |
|  | **PM_2.5_** | | | | **Ozone** | | | |
|  | **High PM_2.5_** | | **Low PM_2.5_** | | **High O_3_** | | **Low O_3_** | |
|  | N= 476,981(50.0%) | | N= 476,970 (50.0%) | | N= 477,013 (50.0%) | | N= 476,938 (50.0%) | |
| Maternal Age (years), M±SD | 28.0 ± 6.4 | | 29.0 ± 6.2 | | 28.5 ± 6.74 | | 28.5 ± 6.3 | |
| n (%) 15-20 | 48,063 | (7.2) | 34,347 | (10) | 40,431 | (8.5) | 41,979 | (8.8) |
| 20-25 | 105,411 | (18.5) | 88,419 | (22.1) | 96,616 | (20.3) | 97,214 | (20.3) |
| 25-30 | 124,373 | (25.7) | 122,380 | (26.1) | 125,585 | (26.3) | 121,168 | (25.4) |
| 30-35 | 115,241 | (27.7) | 132,263 | (24.2) | 124,075 | (26) | 123,429 | (25.9) |
| >35 | 83,893 | (20.9) | 99,561 | (17.6) | 90,306 | (18.9) | 93,148 | (19.5) |
| Race, n (%) |  | |  | |  | |  | |
| White | 246,289 | (51.6) | 259,527 | (54.5) | 280,534 | (58.8) | 225,282 | (47.2) |
| Black | 34,549 | (7.2) | 34,280 | (7.2) | 25,608 | (5.4) | 43,221 | (9.1) |
| Native American/Alaskan Native | 1,121 | (0.2) | 2,089 | (0.4) | 1,190 | (0.2) | 2,020 | (0.4) |
| Asian/ Pacific Islander | 51,530 | (10.8) | 67,758 | (14.2) | 51,153 | (10.7) | 68,135 | (14.3) |
| Other/Unknown | 143,492 | (30.1) | 113,316 | (23.8) | 118,528 | (24.8) | 138,280 | (29) |
| Ethnicity, n (%) |  | |  | |  | |  | |
| Hispanic | 283,376 | (59.4) | 213,735 | (44.8) | 244,200 | (51.2) | 252,911 | (53) |
| Non-Hispanic* | 193,605 | (40.6) | 263,235 | (55.2) | 232,813 | (48.8) | 224,027 | (47) |
| Mother, foreign born, n (%) | 238,188 | (49.9) | 208,633 | (43.8) | 212,249 | (44.5) | 234,572 | (49.2) |
| Maternal education, n (%) |  | |  | |  | |  | |
| Less than high school | 145,596 | (30.5) | 98,379 | (20.6) | 113,244 | (23.7) | 130,731 | (27.4) |
| High school/GED | 127,924 | (26.8) | 111,483 | (23.3) | 120,087 | (25.2) | 119,320 | (25) |
| Some college | 101,241 | (21.2) | 116,298 | (24.4) | 114,430 | (24) | 103,109 | (21.6) |
| College degree or more | 102,220 | (21.4) | 150,810 | (31.6) | 129,252 | (27.1) | 123,778 | (26) |
| Maternal health , n (%) |  | |  | |  | |  | |
| Pre-existing Hypertension | 2,731 | (0.6) | 2,075 | (0.4) | 2,343 | (0.5) | 2,463 | (0.5) |
| Gestational Hypertension | 4,097 | (0.9) | 5,400 | (1.1) | 4,439 | (0.9) | 5,058 | (1.1) |
| Pre-existing Diabetes | 3,959 | (0.8) | 3,823 | (0.8) | 3,749 | (0.8) | 4,033 | (0.8) |
| Gestational Diabetes | 4,240 | (0.9) | 4,211 | (0.9) | 3,907 | (0.8) | 4,544 | (1) |
| Pre-eclampsia | 13,629 | (2.9) | 14,187 | (3) | 12,954 | (2.7) | 14,862 | (3.1) |
| Tobacco use, n(%) |  |  |  |  |  |  |  |  |
| <10 cigs/day | 3,472 | (0.8) | 4,283 | (0.9) | 4,439 | (0.9) | 3,316 | (0.7) |
| 10-20 cigs/day | 975 | (0.2) | 1,721 | (0.3) | 1,721 | (0.4) | 975 | (0.2) |
| >20 cigs/day | 704 | (0.2) | 977 | (0.2) | 1,059 | (0.2) | 622 | (0.1) |
| BMI (pre-pregnancy), M±SD | 25.8 ± 5.9 | | 25.6 ± 6.3 | | 25.6 ± 5.8 | | 25.7 ± 6.4 | |
| Number of Prenatal care visits, M±SD | 12.4 ± 3.7 | | 12.1 ± 3.7 | | 12.3 ± 3.8 | | 12.2 ± 3.7 | |
| Insurance type, n (%) |  |  |  |  |  |  |  |  |
| Medi-Cal | 260,676 | (54.7) | 202,970 | (42.6) | 223,007 | (46.8) | 240,639 | (50.5) |
| Private | 198,298 | (41.6) | 255,623 | (53.6) | 233,476 | (48.9) | 220,445 | (46.2) |
| Birth Year, n (%) |  |  |  |  |  |  |  |  |
| 2007 | 150,285 | (31.5) | 47,990 | (10.1) | 94,440 | (19.8) | 103,835 | (21.8) |
| 2008 | 144,555 | (30.3) | 38,563 | (8.1) | 89,788 | (18.8) | 93,330 | (19.6) |
| 2009 | 113,603 | (23.8) | 65,241 | (13.7) | 93,747 | (19.7) | 85,097 | (17.8) |
| 2010 | 57,528 | (12.1) | 141,240 | (29.6) | 102,874 | (19.7) | 95,894 | (20.1) |
| 2011 | 11,010 | (2.3) | 183,936 | (38.6) | 96,164 | (21.6) | 98,782 | (20.7) |
| Season of conception, n (%) |  |  |  |  |  |  |  |  |
| Fall | 114,431 | (24) | 125,633 | (26.3) | 99,822 | (20.9) | 140,242 | (29.4) |
| Spring | 139,025 | (29.1) | 98,701 | (20.7) | 118,640 | (24.9) | 119,086 | (25) |
| Summer | 130,631 | (27.4) | 104,926 | (22) | 84,046 | (17.6) | 151,511 | (31.8) |
| Winter | 92,894 | (19.5) | 147,710 | (31) | 174,505 | (36.6) | 66,099 | (13.9) |
| Parity, n(%) |  |  |  |  |  |  |  |  |
| nulliparous | 191,350 | (40.1) | 199,831 | (41.9) | 193,775 | (40.6) | 197,406 | (41.4) |
| 1 or more | 285,460 | (59.9) | 277,060 | (58.1) | 283,082 | (59.4) | 279,438 | (58.6) |
| Female sex of infant, n (%) | 232,271 | (48.7) | 232,244 | (48.7) | 232,405 | (48.7) | 232,210 | (48.7) |
