# Supplementary material for: The relationship between air pollutants and maternal socioeconomic factors on preterm birth in California urban counties
Source: J Expo Sci Environ Epidemiol. 2021 Apr 15;31(3):503–13. doi: 10.1038/s41370-021-00323-7 (PMC8134052; doi:10.1038/s41370-021-00323-7)
Supplement: Supplementary file 4 — SupTable 2 [file 41370_2021_323_MOESM4_ESM.docx]

| Table S2. Interaction between maternal exposure to PM_2.5_ and O_3_ and neighborhood census tract-level covariates on preterm birth (aOR^a^±95%CI) | | | | | | | | | |  |  |  |
| --- | --- | --- | --- | --- | --- | --- | --- | --- | --- | --- | --- | --- |
|  | 3-months Pre-pregnancy | | | 1^st^ Trimester | | | 2^rd^ Trimester | | | 3^rd^ Trimester | |  |
| **Exposure to PM_2.5_** | **aOR^a^** | **95% CI** | **p-value** | **aOR^a^** | **95% CI** | **p-value** | **aOR^a^** | **95% CI** | **p-value** | **aOR^a^** | **95% CI** | **p-value** |
| Low HH Income ($) | 1.01 | (0.94, 1.08) | 0.87 | 1.01 | (0.94, 1.09) | 0.72 | 1.01 | (0.94, 1.09) | 0.75 | 0.98 | (0.90, 1.06) | 0.58 |
| Low HS grad (%) | 1.05 | (0.98, 1.13) | 0.16 | 1.02 | (0.95, 1.10) | 0.54 | 1.03 | (0.95, 1.10) | 0.5 | 1.02 | (0.94, 1.11) | 0.67 |
| High Assistance (%) | 1.06 | (0.98, 1.14) | 0.1 | 1 | (0.93, 1.08) | 0.92 | 0.98 | (0.91, 1.06) | 0.65 | 0.97 | (0.89, 1.05) | 0.46 |
| High SNAP (%) | 1.05 | (0.98, 1.13) | 0.16 | 0.99 | (0.92, 1.07) | 0.87 | 1.03 | (0.96, 1.11) | 0.41 | 0.99 | (0.91, 1.08) | 0.9 |
| High Pub (%) | 1.02 | (0.95, 1.09) | 0.63 | 0.99 | (0.92, 1.07) | 0.84 | 1.02 | (0.95, 1.10) | 0.6 | 1.02 | (0.94, 1.11) | 0.58 |
| High SSI (%) | 1.02 | (0.95, 1.09) | 0.62 | 0.99 | (0.92, 1.06) | 0.73 | 1 | (0.93, 1.08) | 0.9 | 0.95 | (0.88, 1.04) | 0.26 |
| High poverty (%) | 1.03 | (0.96, 1.11) | 0.41 | 1.03 | (0.96, 1.11) | 0.45 | 1.02 | (0.94, 1.09) | 0.68 | 0.99 | (0.91, 1.08) | 0.8 |
| High Unemployed (%) | 1.08 | (1.01, 1.17) | 0.03 | 0.99 | (0.92, 1.06) | 0.77 | 0.96 | (0.90, 1.03) | 0.3 | 0.98 | (0.90, 1.06) | 0.57 |
| High GINI index (%) | 0.97 | (0.94, 1.08) | 0.81 | 1.03 | (0.96, 1.10) | 0.44 | 0.99 | (0.92, 1.06) | 0.79 | 0.97 | (0.89, 1.05) | 0.41 |
| **Exposure to O_3_** |  |  |  |  |  |  |  |  |  |  |  |  |
| Low HH Income ($) | 1 | (0.93, 1.07) | 0.9 | 0.99 | (0.92, 1.06) | 0.69 | 1.02 | (0.94, 1.09) | 0.81 | 1.06 | (0.98, 1.16) | 0.16 |
| Low HS grad (%) | 0.99 | (0.92, 1.06) | 0.75 | 0.97 | (0.90, 1.04) | 0.42 | 1.02 | (0.95, 1.09) | 0.63 | 1.13 | (1.04, 1.23) | **0.005** |
| High Assistance (%) | 1 | (0.93, 1.08) | 0.97 | 0.97 | (0.90, 1.04) | 0.44 | 0.99 | (0.92, 1.06) | 0.75 | 1.09 | (1.00, 1.18) | 0.06 |
| High SNAP (%) | 1 | (0.93, 1.07) | 0.92 | 0.99 | (0.92, 1.06) | 0.76 | 1.02 | (0.95, 1.09) | 0.63 | 1.12 | (1.03, 1.22) | 0.01 |
| High Pub (%) | 0.99 | (0.92, 1.06) | 0.75 | 1 | (0.93, 1.08) | 0.99 | 1 | (0.93, 1.08) | 0.96 | 1.13 | (1.04, 1.23) | 0.004 |
| High SSI (%) | 0.98 | (0.91, 1.06) | 0.63 | 1 | (0.93, 1.08) | 0.97 | 0.99 | (0.92, 1.06) | 0.76 | 1.06 | (0.98, 1.15) | 0.17 |
| High poverty (%) | 1 | (0.93, 1.07) | 0.94 | 0.94 | (0.87, 1.01) | 0.07 | 0.99 | (0.92, 1.07) | 0.87 | 1.07 | (0.99, 1.17) | 0.11 |
| High Unemployed (%) | 0.95 | (0.89, 1.02) | 0.2 | 1.02 | (0.95, 1.09) | 0.61 | 1 | (0.93, 1.07) | 0.93 | 1.04 | (0.96, 1.14) | 0.31 |
| High GINI index (%) | 0.98 | (0.91, 1.05) | 0.55 | 1.03 | (0.96, 1.10) | 0.46 | 1.04 | (0.97, 1.12) | 0.28 | 0.99 | (0.92, 1.08) | 0.9 |
|  |  |  |  |  |  |  |  |  |  |  |  |  |
| ^a^ adjusted for season of conception, maternal cigarette use, age, race/ethnicity, education, payment, prenatal visits began in 1^st^ trimester, median used for high/low cut off of census tract-level covariates; P-value is from Wald Chi-Squared test | | | | | | | | | | | | |
| High/Low cutoff is median PM_2.5_= 12.9 *µg/m^3^*, High/Low cutoff for is median O_3_= 39 ppb for the whole pregnancy *(EPA limits are Annual PM_2.5_=12 µg/m^3^, 8-hr max O_3_= 0.070ppm) include the reference category*. Low is the reference category. AOR is for every 1-unit increase in *µg/m^3^* for PM_2.5_ and ppb for *O_3_.* | | | | | | | | | | | | |
| **HH: Household, HS: High school, Assistance: Total assistance, SNAP: Supplemental Nutrition Assistance Program, Pub= Public Assistance (including general assistance and Temporary Assistance to Needy Families (TANF) assistance, SSI: supplemental security income, GINI: GINI index for income inequality | | | | | | | | | | | | |
