# Supplementary material for: The relationship between air pollutants and maternal socioeconomic factors on preterm birth in California urban counties
Source: J Expo Sci Environ Epidemiol. 2021 Apr 15;31(3):503–13. doi: 10.1038/s41370-021-00323-7 (PMC8134052; doi:10.1038/s41370-021-00323-7)
Supplement: Supplementary file 5 — SupTable 3 [file 41370_2021_323_MOESM5_ESM.docx]

| Table S3. Interaction between maternal exposure to PM2.5 and O3 and neighborhood census tract-level covariates on early preterm birth (AOR^a^±95%CI). | | | | | | | | | |  | | | |  |  |
| --- | --- | --- | --- | --- | --- | --- | --- | --- | --- | --- | --- | --- | --- | --- | --- |
|  | 3 months PRE | | | 1^st^ Trimester | | | 2^nd^ Trimester | | | 3^rd^ Trimester | | |  |  |  |
| **Exposure to PM_2.5_** | **AOR^a^** | **95% CI** | **p-value** | **AOR^a^** | **95% CI** | **p-value** | **AOR^a^** | **95% CI** | **p-value** | **AOR^a^** | **95% CI** | **p-value** |  |  |  |
| Low HH Income($) | 0.99 | (0.96, 1.02) | 0.6 | 1 | (0.97, 1.03) | 0.99 | 0.99 | (0.96, 1.02) | 0.47 | 1 | (0.97, 1.03) | 0.82 |  |  |  |
| Low HS grad (%) | 0.97 | (0.94, 0.99) | 0.02 | 0.98 | (0.95 1.01) | 0.24 | 0.98 | (0.95, 1.01) | 0.11 | 0.97 | (0.94, 1.00) | 0.03 |  |  |  |
| High Assistance (%) | 0.99 | (0.97, 1.02) | 0.67 | 1 | (0.97, 1.03) | 0.89 | 0.98 | (0.96, 1.01) | 0.26 | 0.98 | (0.95, 1.01) | 0.15 |  |  |  |
| High SNAP (%) | 0.98 | (0.96, 1.01) | 0.25 | 0.98 | (0.96, 1.01) | 0.3 | 0.98 | (0.95, 1.01) | 0.13 | 0.97 | (0.95, 1.00) | 0.09 |  |  |  |
| High Pub (%) | 0.97 | (0.94, 1.00) | 0.03 | 0.98 | (0.96, 1.01) | 0.26 | 0.97 | (0.94, 0.99) | 0.02 | 0.98 | (0.95, 1.01) | 0.12 |  |  |  |
| High SSI (%) | 0.98 | (0.95, 1.01) | 0.17 | 0.99 | (0.96, 1.02) | 0.59 | 0.98 | (0.95, 1.01) | 0.2 | 0.99 | (0.96, 1.02) | 0.37 |  |  |  |
| High poverty (%) | 0.99 | (0.96, 1.02) | 0.58 | 1 | (0.97, 1.03) | 0.99 | 0.99 | (0.96, 1.02) | 0.13 | 0.99 | (0.96, 1.02) | 0.57 |  |  |  |
| High Unemployed (%) | 0.95 | (0.92, 0.98) | 0.001 | 0.99 | (0.96, 1.01) | 0.3 | 0.97 | (0.94, 0.99) | 0.02 | 0.99 | (0.96, 1.02) | 0.49 |  |  |  |
| High GINI (%) | 1.02 | (0.99, 1.05) | 0.15 | 1.02 | (0.99, 1.05) | 0.14 | 1.02 | (0.99, 1.05) | 0.2 | 1.03 | (1.00, 1.06) | 0.02 |  |  |  |
| **Exposure to O_3_** |  |  |  |  |  |  |  |  |  |  |  |  |  |  |  |
| Low HH Income($) | 1 | (0.97, 1.02) | 0.76 | 0.99 | (0.96, 1.02) | 0.39 | 0.96 | (0.93, 0.99) | 0.005 | 0.98 | (0.95, 1.01) | 0.17 |  |  |  |
| Low HS grad (%) | 1.01 | (0.98, 1.03) | 0.72 | 0.99 | (0.96, 1.02) | 0.55 | 0.94 | (0.91, 0.96) | <0.001 | 0.95 | (0.94, 0.98) | <0.001 |  |  |  |
| High Assistance (%) | 1.03 | (1.00, 1.06) | 0.03 | 1.02 | (1.00, 1.05) | 0.1 | 0.99 | (0.96, 1.01) | 0.34 | 0.99 | (0.96, 1.02) | 0.55 |  |  |  |
| High SNAP (%) | 1.03 | (1.00, 1.06) | 0.07 | 1.02 | (0.99, 1.05) | 0.17 | 0.98 | (0.96, 1.01) | 0.26 | 0.98 | (0.95, 1.00) | 0.5 |  |  |  |
| High Pub (%) | 1.02 | (0.99, 1.05) | 0.21 | 1.02 | (0.99, 1.05) | 0.27 | 0.99 | (0.96, 1.01) | 0.33 | 0.99 | (0.96, 1.01) | 0.55 |  |  |  |
| High SSI (%) | 1.03 | (1.00, 1.06) | 0.07 | 1.03 | (1.00, 1.05) | 0.09 | 0.97 | (0.95, 1.00) | 0.05 | 0.97 | (0.94, 1.00) | 0.03 |  |  |  |
| High poverty (%) | 1.03 | (1.00, 1.06) | 0.04 | 0.98 | (0.96, 1.01) | 0.29 | 0.95 | (0.92, 0.97) | <0.001 | 0.99 | (0.96, 1.01) | 0.32 |  |  |  |
| High Unemployed (%) | 1.02 | (0.99, 1.05) | 0.14 | 1.01 | (0.98, 1.04) | 0.68 | 0.96 | (0.94, 0.99) | 0.009 | 0.98 | (0.95, 1.00) | 0.09 |  |  |  |
| High GINI (%) | 1.06 | (1.03, 1.09) | **<0.001** | 1 | (0.97, 1.03) | 0.96 | 1.01 | (0.99, 1.04) | 0.3 | 1.04 | (1.01, 1.07) | 0.01 |  |  |  |
| ^a^ adjusted for season of conception, maternal cigarette use, age, race/ethnicity, education, payment, prenatal visits began in 1^st^ trimester, median used for high/low cut off of census tract-level covariates; P-value is from Wald Chi-Squared test | | | | | | | | | | | | |  |  |  |
| High/Low cutoff is median PM_2.5_= 12.9 *µg/m^3^*, High/Low cutoff for is median O_3_= 39 ppb for the whole pregnancy *(EPA limits are Annual PM_2.5_=12 µg/m^3^, 8-hr max O_3_= 0.070ppm) include the reference category*. Low is the reference category. AOR is for every 1-unit increase in *µg/m^3^* for PM_2.5_ and ppb for *O_3_.* | | | | | | | | | | | | |  |  |  |
| **HH: Household, HS: High school, Assistance: Total assistance, SNAP: Supplemental Nutrition Assistance Program, Pub= Public Assistance (including general assistance and Temporary Assistance to Needy Families (TANF) assistance, SSI: supplemental security income, GINI: GINI index for income inequality | | | | | | | | | | | | |  |  |  |
|  | | | | | | | | | | | | |  |  |  |
