# Supplementary material for: The relationship between air pollutants and maternal socioeconomic factors on preterm birth in California urban counties
Source: J Expo Sci Environ Epidemiol. 2021 Apr 15;31(3):503–13. doi: 10.1038/s41370-021-00323-7 (PMC8134052; doi:10.1038/s41370-021-00323-7)
Supplement: Supplementary file 6 — SupTable 4 [file 41370_2021_323_MOESM6_ESM.docx]

| Supplemental Table 4. Association between PM_2.5_ and O_3_ and preterm birth by season of conception (aOR^a^±95%CI) | | | | | | | | |  |  |
| --- | --- | --- | --- | --- | --- | --- | --- | --- | --- | --- |
|  |  |  |  |  |  |  |  |  |  |  |
|  | 3 months pre-pregnancy | | 1^st^ Trimester | | 2^nd^ Trimester | | 3^rd^ Trimester | | Whole pregnancy | |
| **Exposure to PM_2.5_** | aOR^b^ | 95% CI | aOR^b^ | 95% CI | aOR^b^ | 95% CI | aOR^b^ | 95% CI | aOR^b^ | 95% CI |
| Winter | 1.11 | (1.08, 1.51) | 1.13 | (1.10, 1.17) | 1.12 | (1.08, 1.15) | 0.92 | (0.89, 0.95) | 1.04 | (1.01, 1.07) |
| Spring | 0.96 | (0.93, 0.99) | 0.9 | (0.88, 0.93) | 0.99 | (0.96, 1.02) | 0.97 | (0.94, 1.00) | 0.85 | (0.82, 0.87) |
| Summer | 1.06 | (1.03, 1.09) | 1.08 | (1.04, 1.11) | 1.09 | (1.06, 1.13) | 1.41 | (1.37, 1.45) | 1.37 | (1.33, 1.41) |
| Fall | 1.08 | (1.05, 1.11) | 1.1 | (1.07, 1.14) | 1.09 | (1.06, 1.13) | 0.95 | (0.92, 0.97) | 1.2 | (1.17, 1.24) |
| **Exposure to O_3_** |  |  |  |  |  |  |  |  |  |  |
| Winter | 0.94 | (0.85, 1.03) | 1.1 | (1.07, 1.14) | 1.14 | (1.09, 1.19) | 1.65 | (1.60, 1.71) | 1.31 | (1.26, 1.35) |
| Spring | 1.13 | (1.10, 1.17) | 1.17 | (1.12, 1.22) | 1.03 | (1.00, 1.06) | 4.48 | (4.24, 4.74) | 1.67 | (1.62, 1.72) |
| Summer | 1.17 | (1.12, 1.22) | 1.07 | (1.03, 1.10) | 0.96 | (0.87, 1.06) | 0.49 | (0.47, .050) | 0.78 | (0.76, 0.81) |
| Fall | 1.06 | (1.03, 1.09) | 0.91 | (0.83, 1.01) | 1.04 | (1.01, 1.07) | 1.32 | (1.26, 1.38) | 0.72 | (0.70, 0.74) |
|  |  |  |  |  |  |  |  |  |  |  |
| *High/Low cutoff is median PM_2.5_= 12.9, High/Low cutoff for is median O_3_= 39 ppb for the whole pregnancy *(EPA limits are Annual PM_2.5_=12 µg/m^3^, 8-hr max O_3_= 0.070ppm) include the reference category*. High is the reference category | | | | | | | | | | |
| ^a^ adjusted for maternal cigarette use, age, race/ethnicity, education, payment, prenatal visits began in 1^st^ trimester | | | | | | | | |  |  |
