# Supplementary material for: The relationship between air pollutants and maternal socioeconomic factors on preterm birth in California urban counties
Source: J Expo Sci Environ Epidemiol. 2021 Apr 15;31(3):503–13. doi: 10.1038/s41370-021-00323-7 (PMC8134052; doi:10.1038/s41370-021-00323-7)
Supplement: Supplementary file 7 — SupTable 5 [file 41370_2021_323_MOESM7_ESM.docx]

| Supplemental Table 5. Mixed-effects model for association between preterm birth and air pollution | | |
| --- | --- | --- |
|  |  |  |
|  | **Preterm Birth (<37 wks)** | |
|  | N= 87,495/953,951 | |
|  | **Adjusted^a^** | |
| **Exposure to PM_2.5_** | aOR^a^ | (95% CI) |
| 3 Months Pre-pregnancy | 1.00 | (0.99, 1.01) |
| 1^st^ Trimester | 1.00 | (0.99, 1.01) |
| 2^nd^ Trimester | 1.01 | (1.00, 1.01) |
| 3^rd^ Trimester | 0.98 | (0.97, 0.99) |
| Whole Pregnancy | 1.02 | (1.01, 1.02) |
| **Exposure to O_3_** |  |  |
| 3 Months Pre-pregnancy | 1.03 | (1.02, 1.04) |
| 1^st^ Trimester | 1.02 | (1.01, 1.02) |
| 2^nd^ Trimester | 1.00 | (0.99, 1.01) |
| 3^rd^ Trimester | 1.02 | (1.01, 1.02) |
| Whole Pregnancy | 1.00 | (1.00, 1.01) |
|  |  |  |
| *High/Low cutoff is median PM_2.5_= 12.9, High/Low cutoff for is median O_3_= 39 ppb for the whole pregnancy *(EPA limits are Annual PM_2.5_=12 µg/m^3^, 8-hr max O_3_= 0.070ppm) include the reference category*. High is the reference category | | |
| ^a^ adjusted for race/ethnicity, education, MediCal, year of birth, census-tract | | |
