# Supplementary material for: The relationship between air pollutants and maternal socioeconomic factors on preterm birth in California urban counties
Source: J Expo Sci Environ Epidemiol. 2021 Apr 15;31(3):503–13. doi: 10.1038/s41370-021-00323-7 (PMC8134052; doi:10.1038/s41370-021-00323-7)
Supplement: Supplementary file 8 — SupTable 6 [file 41370_2021_323_MOESM8_ESM.docx]

| Supplemental Table 6. Fixed cohort sensitivity analysis for association between preterm birth and air pollution** | | | | |
| --- | --- | --- | --- | --- |
|  |  |  |  |  |
|  | **Preterm Birth (<37 wks)** | | **Preterm Birth (<37 wks)** | |
|  | N= 87,495/953,951 | | N= 81,765/878,121 | |
|  | **ORIGINAL Adjusted^a^** | | **FIXED COHORT Adjusted^a^** | |
| **Exposure to PM_2.5_** | aOR^a^ | (95% CI) | aOR^a^ | (95% CI) |
| 3 Months Pre | 1.06 | (1.04, 1.08) | 0.96 | (0.94, 0.97) |
| 1^st^ Trimester | 1.05 | (1.04, 1.07) | 0.95 | (0.93, 0.97) |
| 2^nd^ Trimester | 1.08 | (1.06, 1.10) | 0.96 | (0.95, 0.98) |
| 3^rd^ Trimester | 1.05 | (1.03, 1.06) | 0.95 | (0.93, 0.96) |
| Whole Pregnancy | 1.10 | (1.09, 1.12) | 1.00 | (0.99, 1.02) |
| **Exposure to O_3_** |  |  |  |  |
| 3 Months Pre | 1.10 | (1.08, 1.12) | 1.08 | (1.06, 1.10) |
| 1^st^ Trimester | 1.09 | (1.07, 1.11) | 1.09 | (1.07, 1.11) |
| 2^nd^ Trimester | 1.05 | (1.03, 1.07) | 1.07 | (1.05, 1.09) |
| 3^rd^ Trimester | 1.06 | (1.04, 1.08) | 1.13 | (1.10, 1.15) |
| Whole Pregnancy | 1.05 | (1.03, 1.06) | 1.06 | (1.04, 1.07) |
|  |  |  |  |  |
| *High/Low cutoff is median PM_2.5_= 12.9, High/Low cutoff for is median O_3_= 39 ppb for the whole pregnancy *(EPA limits are Annual PM_2.5_=12 µg/m^3^, 8-hr max O_3_= 0.070ppm) include the reference category*. High is the reference category | | | | |
| ^a^ adjusted for season of conception, maternal cigarette use, age, race/ethnicity, education, payment, prenatal visits began in 1^st^ trimester, year of birth | | | | |
| **Fixed cohort created by removing births with conception dates 20 weeks or more before the beginning of the study period and 42 weeks or less before the end of the study period | | | | |
